# Low-level language processing in brain-injured patients

**DOI:** 10.1093/braincomms/fcad094

**Published:** 2023-03-25

**Authors:** Parul Jain, Mary M Conte, Henning U Voss, Jonathan D Victor, Nicholas D Schiff

**Affiliations:** Weill Cornell Graduate School of Medical Sciences, New York, NY 10065, USA; Feil Family Brain and Mind Research Institute, Weill Cornell Medical College, New York, NY 10065, USA; College of Human Ecology, Cornell University, Ithaca, NY 14850, USA; Department of Radiology, Weill Cornell Medicine, New York, NY 10065, USA; Feil Family Brain and Mind Research Institute, Weill Cornell Medical College, New York, NY 10065, USA; Department of Neurology, New York Presbyterian Hospital, New York, NY 10065, USA; The Rockefeller University Hospital, New York, NY 10065, USA; Feil Family Brain and Mind Research Institute, Weill Cornell Medical College, New York, NY 10065, USA; Department of Neurology, New York Presbyterian Hospital, New York, NY 10065, USA; The Rockefeller University Hospital, New York, NY 10065, USA

**Keywords:** disorders of consciousness, phoneme, speech envelope

## Abstract

Assessing cognitive function—especially language processing—in severely brain-injured patients is critical for prognostication, care, and development of communication devices (e.g. brain–computer interfaces). In patients with diminished motor function, language processing has been probed using EEG measures of command-following in motor imagery tasks. While such tests eliminate the need for motor response, they require sustained attention. However, passive listening tasks, with an EEG response measure can reduce both motor and attentional demands. These considerations motivated the development of two assays of low-level language processing—identification of differential phoneme-class responses and tracking of the natural speech envelope. This cross-sectional study looks at a cohort of 26 severely brain-injured patient subjects and 10 healthy controls. Patients’ level of function was assessed via the coma recovery scale–revised at the bedside. Patients were also tested for command-following via EEG and/or MRI assays of motor imagery. For the present investigation, EEG was recorded while presenting a 148 s audio clip of *Alice in Wonderland*. Time-locked EEG responses to phoneme classes were extracted and compared to determine a differential phoneme-class response. Tracking of the natural speech envelope was assessed from the same recordings by cross-correlating the EEG response with the speech envelope. In healthy controls, the dynamics of the two measures were temporally similar but spatially different: a central parieto-occipital component of differential phoneme-class response was absent in the natural speech envelope response. The differential phoneme-class response was present in all patient subjects, including the six classified as vegetative state/unresponsive wakefulness syndrome by behavioural assessment. However, patient subjects with evidence of language processing either by behavioural assessment or motor imagery tests had an early bilateral response in the first 50 ms that was lacking in patient subjects without any evidence of language processing. The natural speech envelope tracking response was also present in all patient subjects and responses in the first 100 ms distinguished patient subjects with evidence of language processing. Specifically, patient subjects with evidence of language processing had a more global response in the first 100 ms whereas those without evidence of language processing had a frontopolar response in that period. In summary, we developed two passive EEG-based methods to probe low-level language processing in severely brain-injured patients. In our cohort, both assays showed a difference between patient subjects with evidence of command-following and those with no evidence of command-following: a more prominent early bilateral response component.

## Teaser

Jain et al. find evidence of the processing of phonemes, the basic auditory unit of language, in subjects with disorders of consciousness after severe brain injury, including those without overt signs of language comprehension. They identify a specific response pattern that distinguishes those subjects with and without any evidence of language comprehension.

## Graphical Abstract

**Graphical abstract:**
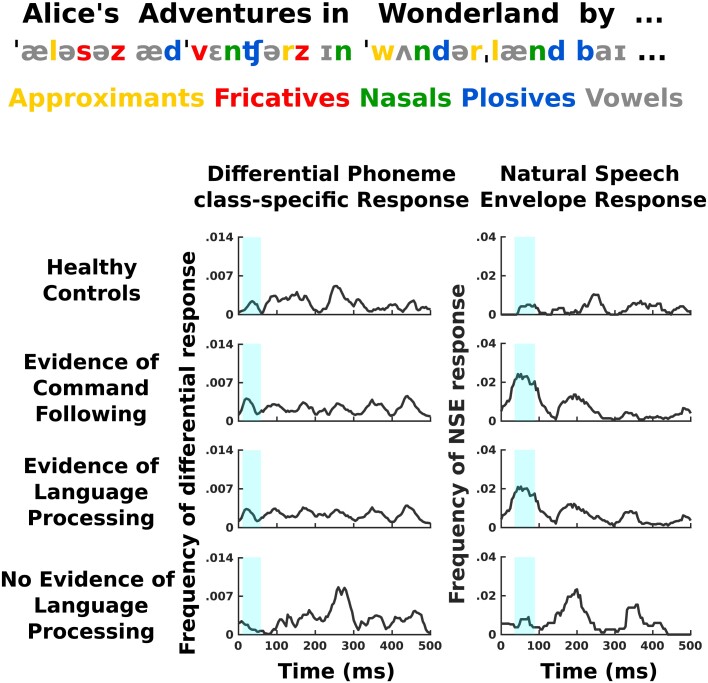

## Introduction

Severe brain injury often results in disorders of consciousness (DoC^1^), with impaired cognitive function including deficits of motor function, executive function, and most importantly, the ability to communicate. Since communication involves the processing of language at multiple levels, loss of the ability to communicate can stem from a loss of processing at any of the levels. Current evaluations of cognitive function in this patient population assess higher-order cognitive skills and are not designed to probe language processing at lower levels.^2^ This study addresses the question of feasibility and informativeness of lower-level language processing studies for the assessment of function in DoC patients.

Assessment of cognitive function can be especially difficult in patients with severe motor deficits. Specifically, behavioural assessments of the ability to follow commands, including standardized batteries such as the coma recovery scale revised (CRS-R)^3^ or Glasgow coma scale,^4^ can overlook residual linguistic function, since they rely on the patient's ability to produce an overt motor response to commands.^5^ In particular, such assessments will not identify patients with preserved cognitive skills but severely impaired motor function [cognitive motor dissociation (CMD)].^6^

To bypass the need for motor response, auxiliary tests of linguistic function have been developed.^7^ These tests record EEG^8,9^ or fMRI^10,11^ signals while the patient is asked to imagine performing an action, such as ‘imagine swimming’. Reproducible changes in the EEG or the blood-oxygen-level-dependent response linked to these commands serve to identify patients with CMD.^9,10,12^ However, since these tests rely on the ability to imagine performing a motor task repeatedly over several minutes, they also require sustained attention.

Thus, while current EEG and fMRI-based tests solve the problem of requiring a motor response, they may overlook patients with impaired executive function. Additionally, current methods are not designed to identify patients who have retained only very basic elements of language processing—such as phonemic processing—but without evidence of a communication channel.

To fill this gap, we used two EEG-based methods for assessing lower-level language processing that avoid the need for motor responses or sustained attention. These methods assay language processing at two levels: differentiation of phoneme classes and tracking of the natural speech envelope (NSE). Phonemes are the basic units of a language that come together to form words.^13^ NSE tracks the pressure changes due to speech sounds; these changes correspond to transitions between phonemes.^14^ Both methods are considered passive listening tasks, in the sense that they do not explicitly ask the subject to engage or respond. The existence of differential EEG responses to phoneme classes, classified based on the manner of articulation, has been demonstrated in normal subjects,^15^ but the approach has not yet been applied to clinical populations. The feasibility of NSE tracking studies in severely brain-injured patients has been established by previous studies.^16^

Here, we seek to identify signals in EEG corresponding to differential responses to phoneme classes and NSE tracking in a passive listening paradigm. This complements recent studies assessing language processing in severe brain-injury patients, which looked at the processing of words, phrases, and sentences in short, structured stimuli.^17,18^ Thus, this work expands the technology for multilevel language processing assessments in this population.

## Methods

### Participants

Ten healthy controls (HC) with no history of neurological disease participated in this study (mean age 37.9 years, 5 male). Patient subjects (PS, *n* = 26) chosen for this study were drawn from a convenience sample enrolled in multimodal imaging and behavioural studies of the natural history of recovery from severe brain injury. Key inclusion/exclusion criteria were age at the time of the study (between 18 and 75), language competence (spoke English before injury), and absence of a premorbid neuropsychiatric history (see Curley *et al.*^19^ for detailed inclusion/exclusion criteria). Data from two subjects from each group were not analysed due to excessive myogenic artefacts in the EEG. Details about the remaining 8 HC and 24 PS are provided in Table 1. Patients were typically admitted for 2–4 day EEG studies to either New York Presbyterian or The Rockefeller University Hospital. In patients without MRI contraindications, fMRI studies were obtained the same day before hospital admission. The protocol was approved by the institutional review boards of Weill Cornell Medicine and The Rockefeller University. HCs provided written consent. For patient subjects, written consent was provided by their legally authorized representatives.

**Table 1:** Subject details

Subject	Sex	Age at injury	Aetiology of injury	Age at study	Bedside classification	Command-following	
						EEG	fMRI
PS01^a^	F	12	TBI/Hypoxia	23	MCS−	neg.	neg.
				26	MCS+	pos.	neg.
PS02^a^	M	15	TBI	22	MCS−	neg.	pos.
PS03^a^	M	16	TBI	25	VS	pos.	pos.
PS04	F	16	TBI	22	VS	neg.	neg.
PS05	M	16	TBI	21	MCS+	neg.	neg.
PS06^a^	M	17	TBI	47	MCS–	pos.	not done
PS07^a^	M	17	Prob. Vasc. w. Ischaemia	21	MCS+	pos.	neg.
PS08^a^	F	17	TBI	19	MCS+	pos.	neg.
				20	eMCS	pos.	neg.
PS09^a^	M	19	TBI	25	MCS+	pos.	pos.
PS10^a^	M	20	Trauma/Hem., Stroke	32	eMCS	pos.	pos.
PS11^a^	M	21	TBI	27	eMCS	pos.	neg.
				28	MCS+	pos.	pos.
PS12^a^	M	21	TBI	22	MCS+	pos.	pos.
				24	MCS+	neg.	neg.
PS13^a^	M	22	TBI	26	MCS−	pos.	pos.
PS14	F	23	TBI	26	VS	pos.	neg.
PS15^a^	M	24	TBI	27	MCS–	neg.	neg.
PS16	F	32	AVM	34	MCS−	neg.	neg.
PS17	F	36	Hypoxia	38	VS	pos.	not done
PS18	M	40	TBI/Hypoxia	49	MCS+	neg.	pos.
PS19	F	42	Toxic Encephalopathy	44	MCS+	neg.	neg.
PS20^a^	F	51	Anoxia	52	eMCS	pos.	neg.
				52	eMCS	pos.	neg.
				53	eMCS	pos.	neg.
PS21^a^	M	53	SAH	56	eMCS	pos.	not done
PS22	M	56	CATH/Anoxia	59	VS	neg.	neg.
PS23	M	56	CATH/Anoxia	58	eMCS	neg.	not done
				59	eMCS	neg.	neg.
PS24	F	74	CATH/Anoxia	77	VS	neg.	not done
HCs							
HC01*	M			23	HC		
HC02*	F			26	HC		
HC03*	M			26	HC		
HC04	M			35	HC		
HC05	F			36	HC		
HC06*	F			41	HC		
HC07*	M			51	HC		
HC08	F			55	HC		

Patient subjects with multiple visits have multiple rows in the last four columns. HCs with two visits approximately 6 months apart are indicated by an asterisk in the first column. Aetiologies: TBI, traumatic brain injury; AVM, arteriovenous malformation; SAH, subarachnoid haemorrhage; CATH, cardiac arrest treated with therapeutic hypothermia. Patient subjects previously reported in Curley *et al.*^19^ are indicated by a (only the first visit of PS12 and PS20 is reported there). All HCs were previously reported in Curley *et al.*^19^

Patient subjects (PS) were classified into DoC categories [vegetative state (VS)/unresponsive wakefulness syndrome (UWS), MCS−, MCS+, and eMCS] based on quantitative behavioural assessments using the CRS-R.^3,20^ The CRS-R was repeatedly administered by an investigator (author NDS) at each admission, typically at least once per day, and the highest CRS-R score obtained during that visit was used for analysis. The first CRS-R was administered within 24 hours of the hospital admission (i.e. within 24 hours of the onset of EEG recording, and within 24 hours of the fMRI studies if done). For PS admitted for multiple visits (indicated by multiple rows for the PS in Table 1), DoC categorization was assessed separately at each visit. Behavioural classifications were usually consistent across evaluations and are reported in Table 1. This behavioural categorization resulted in the following DoC distribution: VS/UWS, 8; MCS−, 7; MCS+, 10; eMCS, 6.

To identify patient subjects (PS) with evidence of covert command-following, they were tested using the EEG and fMRI paradigms described in Curley *et al.*^19^ for EEG and Forgacs *et al.*^8^ and Bardin *et al.*^12^ for fMRI. Results from 15 of our patient subjects were previously reported in (Curley *et al*.;^19^ see also Table 1). For all PS in our cohort, a single investigator (author HUV) conducted the fMRI studies and analyses. For the 11 PS not included in Curley *et al.*,^19^ two investigators (authors PJ and MMC) analysed the EEG using the methods described therein. Command-following results obtained via EEG and fMRI are reported in Table 1. Patient subjects who showed evidence of command-following either by EEG or fMRI criteria are designated here as CF+ (16 of the 24 PS demonstrated command-following during at least one visit). Evidence of command-following (via CRS-R, EEG or fMRI) was used as a proxy for evidence of language processing, allowing us to further categorize patients for the presence or absence of language processing as LP + (if positive on any of those three measures) or LP− (if not). This further categorization resulted in the following PS distribution: CF+, 16; LP+, 19; LP−, 8.

For some patients (PS01, PS08, PS11), classification based on CRS-R alone, or further categorization based on auxiliary methods (EEG, fMRI) changed across visits, as indicated in the last three columns of Table 1. Figures report the number of subjects (n_s_) and the number of visits (n_v_) for each PS category.

### Data collection

All HCs and patient subjects underwent 24-hour video-EEG recordings. Five of the eight HC participated in two 24-hour studies, 6 months apart (indicated by * in the first column of Table 1). PS participated in 48–72-hour in-patient studies (up to three visits per PS). The auditory stimuli were presented 2–5 times over the course of each visit via disposable earbud headphones. Raw EEG tracings were visually inspected for motion artefacts and only trials with no more than approximately 15 s of artefact (these segments were moved at a later stage) were retained for further analysis. Four subjects [2 HC; 1 VS/UWS (LP−); 1 MCS+ (LP−)] did not have any artefact-free trials and were not considered for further analysis. Characteristics of the remaining subjects (24 PS, 8 HC) are shown in Table 1. Across these subjects, an average of 15% of datasets per subject were rejected because of artefacts.

### Stimulus

The audio passage was an excerpt from the first chapter of *Alice’s Adventures in Wonderland* by Lewis Carroll, read by Kristen McQuillin, available at www.librivox.org. We used a 2 min 38 s long segment, starting at 10 s into the original 10 min 40 s file. The audio passage was sampled at 44.1 kHz. Volume was adjusted to a comfortable level determined by the examiner at the bedside.

### Audio annotation

The audio file was annotated for phonemes using the P2FA toolkit^21^ which provided the start and end times of each phoneme. Phonemes were then grouped into five classes based on the manner of articulation: approximants (A), fricatives (F), nasals (N), plosives (P), and vowels (V). An example of phoneme encoding of the stimulus is shown in Supplementary Fig. 1, with colours marking the different phoneme-classes. The passage had the following phoneme distribution: A, 163; F, 264; N, 147; P, 355; V, 617.

### Data recording

**Figure 1:**
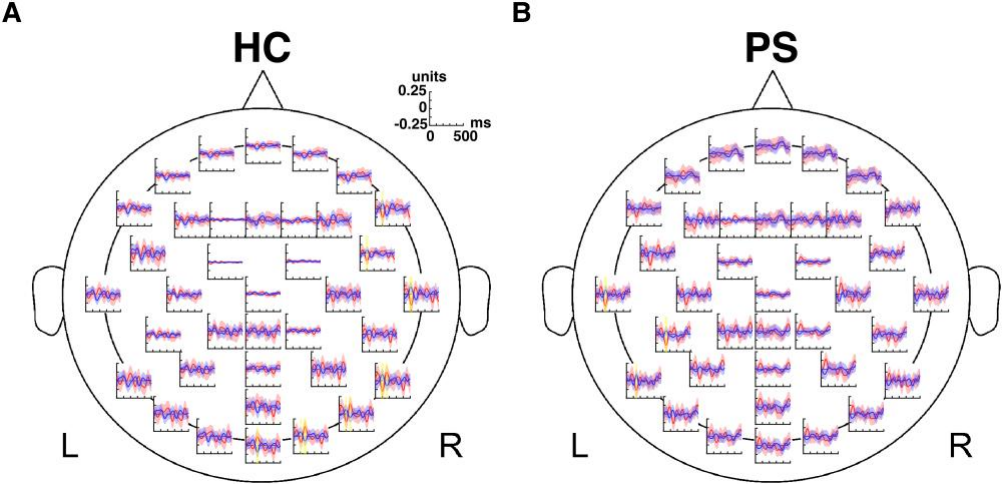
**Phoneme-class response of a single phoneme-class pair for a healthy control (HC) and a patient subject (PS).** The average EEG tracings for plosives (red) and vowels (blue) for a single trial are shown for a healthy control (HC01) in **A** and a PS11 in **B**. For each channel, time points with a significant difference in responses to the two phoneme classes are marked in yellow (Wilcoxon ranksum test; two-tailed, *P* < 0.05 with false-discovery rate (FDR) correction; see Analysis section for details). Natural speech envelope (NSE) tracking responses for the same trial are shown in Supplementary Fig. 2.

The EEG was recorded using 37 electrodes (Nihon Kohden collodion-pasted Ag/AgCl cup electrodes, 1.5 mm), placed according to the International 10–20 System,^22^ with 18 additional electrodes. This recording montage is shown in Fig. 1A and B and Supplementary Fig. 2A and B. Recordings were made using the Natus XLTEK EEG data acquisition system (Natus Medical, San Carlos, CA). The EEG reference electrode was FCz for all recordings. Impedances were measured and maintained at or below 5 kΩ per channel. Signals were amplified and digitized at 250 Hz using an anti-aliasing high pass filter with a corner frequency of 0.4 Hz times the digitization rate. One patient subject (PS04) was tested using 21 electrodes with only two additional electrodes due to a small head size (Table 1).

Presentation software (Neurobehavioral Systems, Inc., Albany, CA) was used for audio delivery. Time-locking of the audio stimulus to the EEG was accomplished by recording the audio signal using an auxiliary channel of the EEG system.

### Data preprocessing

Data were imported into MATLAB R2018b (MathWorks, Natick, MA), notch-filtered to remove 60 Hz noise and then band-pass filtered to 2–15 Hz, based on the approach of Khalighinejad *et al.*^15^ Each channel was standardized to zero mean and unit variance. Data were visually inspected to identify and remove segments with motion artefacts from the analysis, resulting in the removal of 4.13% of the phonemes per trial on average.

### Analysis

We analysed the EEG data for two levels of speech processing: differential responses to phonemic classes (DPR) and tracking of NSE. Several specifics of the method were shaped by the needs of the DPR analysis, and we adapted the NSE analysis to be comparable. We therefore first explain the analysis pipeline for DPR and then for NSE. The analyses were carried out in parallel on each trial and then averaged across trials and/or patient categories in a similar manner.

For DPR analysis, the artefact-free portions of the record were cut into 500 ms segments beginning at each phoneme onset, based on the annotation markers identified from the audio clip (see Audio annotation). Since each phoneme lasts 79.5 ms on average (range 30–320 ms), there was considerable overlap of these segments. Any segment with an amplitude over 10 standard deviations was removed as artefact, resulting in a further loss of 0.19% of the original phoneme responses on average.

We compared the responses to the five phoneme classes in 10 pairwise comparisons, via the Wilcoxon rank-sum test (two-tailed) applied to each time point in the 500 ms segments. This analysis was done separately for each channel. For each channel, the difference was considered statistically significant if the false discovery rate (FDR) corrected *P*-value was <0.05, using the Benjamini–Hochberg^23^ method across the time points. That is, for random data, there will be, on average, 0.05 false-positive points per channel. An alternate analysis in which the FDR correction was done across all channels and time points gave comparable results.

NSE analysis was done as described in Ref. 16 with modifications to make it comparable to DPR analysis. Specifically, after the above preprocessing to remove artefacts, filter, and scale, the EEG was cut into 2-second non-overlapping segments. These segments were cross-correlated with the audio signal, with a maximum lag of 500 ms. The audio signal used for cross-correlation analysis was the average of the binaural input signal. To determine the lags at which the cross-correlation is statistically significant, we generated a null dataset by randomly shuffling the 2-second segments of the EEG response and audio input 10,000 times and calculated the cross-correlation values for the shuffled data. As for the DPR, the criterion for statistical significance was *P* < 0.05 after FDR correction across time points within a channel.

Results for both analyses were combined across trials within a subject, and then across subjects. For the across-subject average, each subject was given equal weight, independent of the number of trials.

## Results

The goal of this study is to identify and characterize signals in the EEG that correspond to differential phoneme-class responses (DPR), alongside a parallel analysis of the EEG response to the NSE. We begin by illustrating the DPR analysis by following sample datasets from a healthy control (HC), and a patient subject (PS); Supplementary Figs 2 and 3 show the corresponding steps for the NSE analysis. We then describe the findings across the HC and PS populations. Finally, we stratify PS according to behavioural assessment for level of function (via the CRS-R) and according to whether they had EEG or fMRI evidence of command-following (see Methods section).

**Figure 2:**
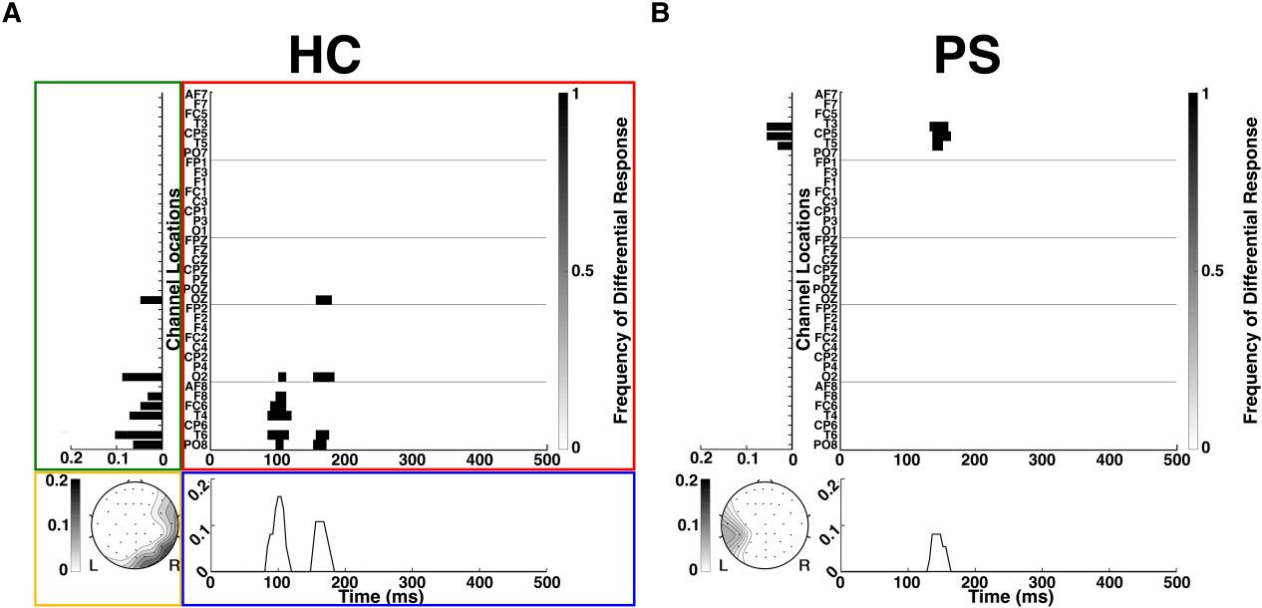
**Differential phoneme-class responses (DPR) in a single phoneme-class pair for a healthy control (HC) and a patient subject (PS).** The data in Fig. 1 is presented in four views, delineated on panel (**A**) (for HC) with coloured boxes: the red box shows the channels and time-points with a significant difference in responses for the two phoneme classes; the blue box shows the frequency of significant differences over time; the green and yellow boxes show the frequency of significant differences in responses for channels as a bar plot and as a contour map over the scalp. The chance response level is ∼0.0004, corresponding to the two-tailed Wilcoxon ranksum test, *P* < 0.05 with false-discovery rate (FDR) correction; see Analysis section for details. Panel (**B**) shows parallel results for PS shown in Fig. 1.

**Figure 3:**
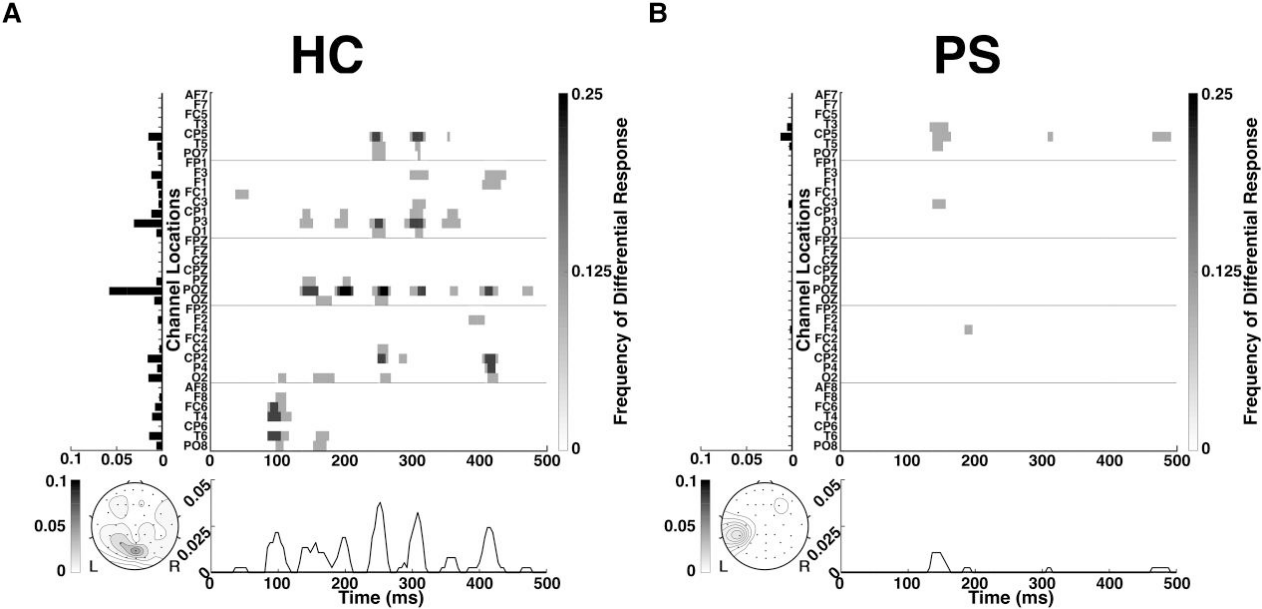
**Differential phoneme-class responses (DPR) in a single trial for a healthy control (HC) and a patient subject (PS).** For the (**A**) HC and (**B**) PS shown in Figs 1 and 2, the DPR is averaged across all phoneme pairs (five phonemes, ten pairs). The chance response level is ∼0.0004, corresponding to the two-tailed Wilcoxon ranksum test, *P* < 0.05 with FDR correction; see Analysis section for details. Natural speech envelope (NSE) tracking responses for the same trial are shown in Supplementary Fig. 3.

Figures 1–3 track two example datasets through the DPR processing pipeline. As detailed in Methods section, we extracted 500-ms segments, time-locked to the onset of each phoneme and averaged them within predefined phoneme classes. The results of this average for a single repeat are shown in Fig. 1 for two phoneme classes: plosives (red) and vowels (blue). Next, to determine the statistical significance of these differences at each time point, we applied the Wilcoxon ranksum test to the distributions of normalized signal amplitudes for the two classes. The time points where the difference in responses remains statistically significant following an FDR correction within each tracing (*P* < 0.05) are shown in yellow. As the panels show, in each dataset, differences were found at multiple scalp locations and time points.

Figure 2 summarizes the above analysis as a heatmap (red box for HC in panel A). Each dark spot in the DPR corresponds to a scalp location and time at which a significant difference in the responses to the two phoneme classes was identified. This corresponds to the significant timepoints marked in yellow in Fig. 1. The heatmap thus highlights the topographic and temporal aspects of the differential responses, further emphasized individually by the average temporal (blue box for HC in panel A) and spatial (green and yellow boxes for HC in panel A) responses. Temporally, the responses for both HC and PS are observed in 100–200 ms, with two peaks for HC but a single peak for PS. Spatially, the responses are right-lateralized for HC with an occipital component and left lateralized for PS.

Figure 3 combines these heatmaps across all 10 phoneme pairs. These averaged DPR plots show differential responses at additional scalp locations and time points compared to the plots for a single phoneme pair (Fig. 2), indicating that the pattern of differential phoneme responses depends on the specific pair of phoneme classes. No single scalp location or time point has differential responses to all phoneme pairs, but they do overlap partially. This partial overlap of the differential responses to different phoneme pairs is indicated by the range of gray levels in Fig. 3: darker marks indicate locations and times at which differential responses to multiple pairs were seen. With averaging across all phoneme pairs, the HC response has multiple peaks both spatially and temporally; the PS primarily has a single left-lateralized peak that is maximal at approximately 150 ms.

**Figure 4:**
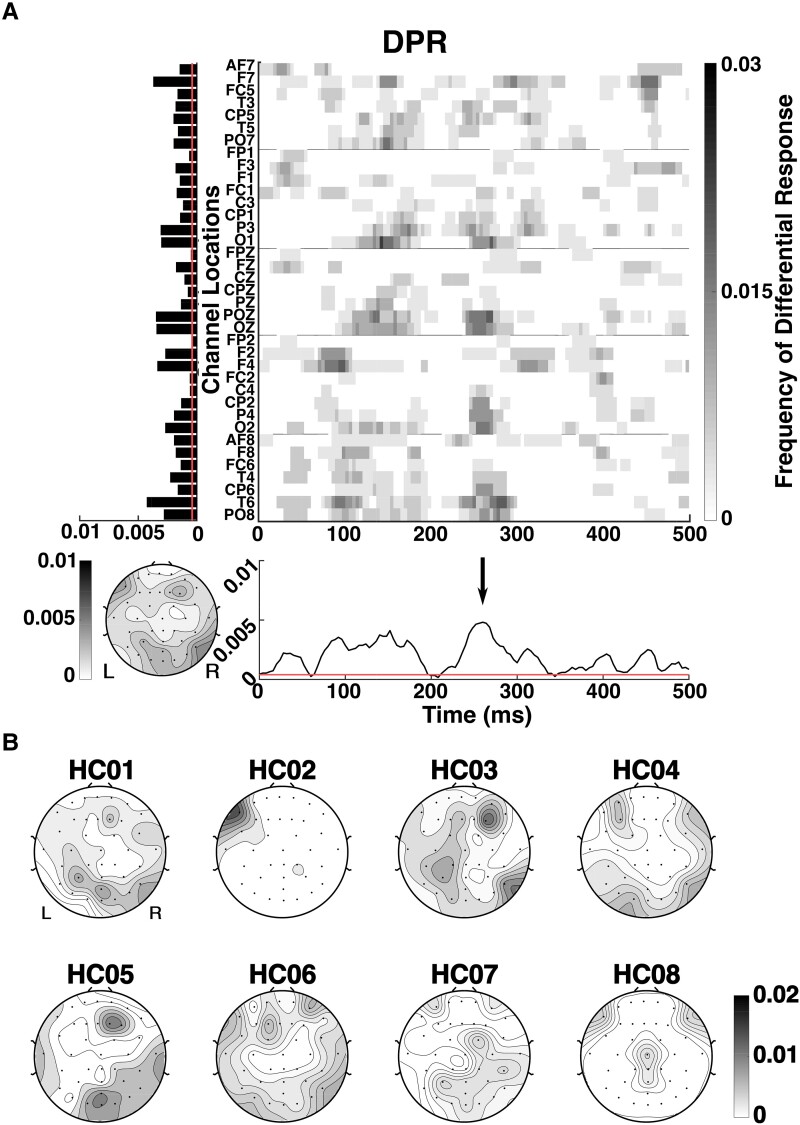
**Average differential phoneme-class response (DPR) across HCs and spatial distribution of DPR for individual HCs.** Responses for each subject are averaged across phoneme pairs and trials. (**A**) Red lines on the time course plot and spatial bar plot mark the chance level (∼0.0004), corresponding to the two-tailed Wilcoxon ranksum test, *P* < 0.05 with false-discovery rate (FDR) correction; see Analysis section for details. Arrow in timecourse plot marks response at 250 ms after phoneme start. (**B**) Spatial distribution of DPR for individual HCs. Ordinates and colorbars indicate the frequency of differential responses. Other graphical conventions as in Fig. 3.

Figure 4A shows the characteristics of the DPR averaged across the HC population. We first consider the topography. The composite map has multiple peaks: left frontal, central parieto-occipital, right temporal, and right frontal. Data from individual subjects (Fig. 4B) shows substantial intersubject variability: no single subject manifests all of the peaks in the average. However, each of the peaks in the cross-subject average is observed in more than one HC. For example, the left frontal peak is seen in subjects HC02, HC04, HC06 and HC08; the right temporal peak is seen in subjects HC01, HC03, HC04, HC05 and HC06; and the right frontal peak is seen in subjects HC01, HC03 and HC05. Of note, there is a prominent midline parieto-occipital peak in subjects HC01, HC05 and HC07, and subjects HC03, HC04 and HC06 also have activity in this area. As mentioned in the Discussion below, each of these peaks is likely to correspond to cortical areas involved in language processing.

In the temporal domain, Fig. 4A shows that the differential phoneme responses in HC subjects occur throughout the 500 ms interval after phoneme onset, consistent with the findings of Khalighinejad *et al*.^15^ There is a dominant feature around 250 ms (indicated by an arrow in Fig. 4). Intersubject variability will be discussed below.

**Figure 5:**
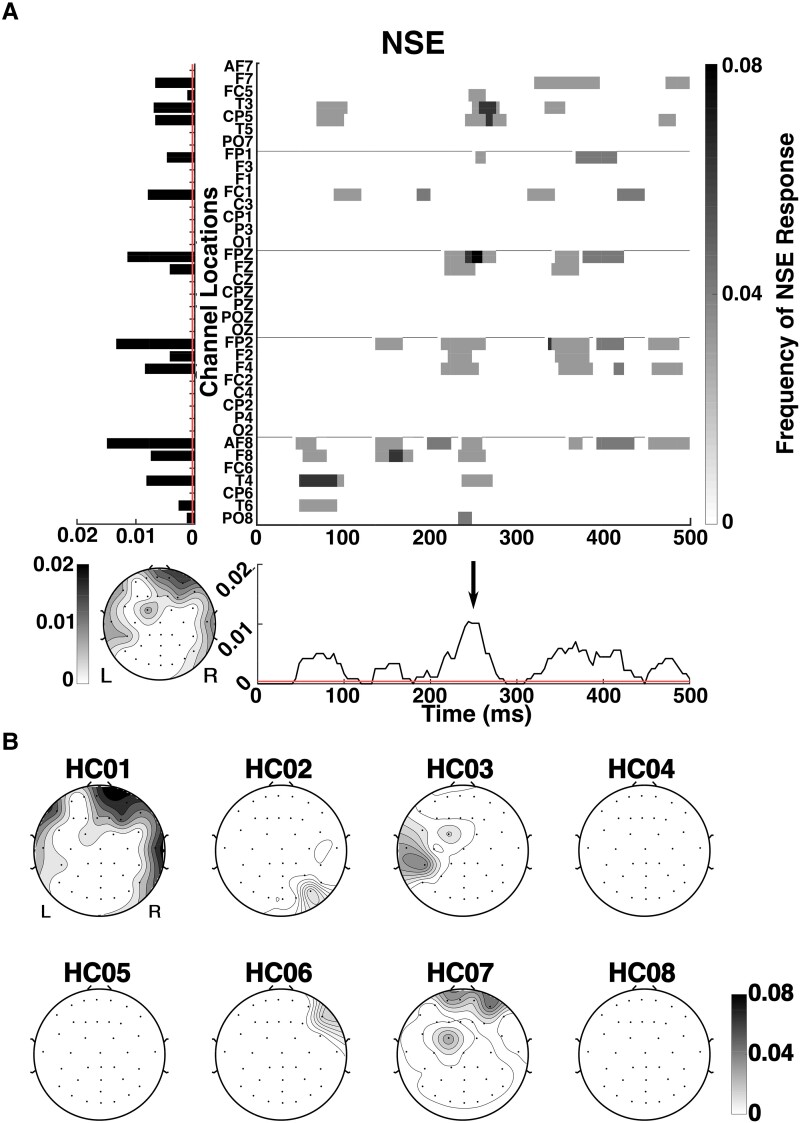
**Average natural speech envelope (NSE) response across HCs and spatial distribution of NSE responses for individual HCs.** Responses for each subject are averaged across trials. Ordinates and colorbars indicate the frequency of an NSE response, measured as significant cross-correlation between EEG response and speech envelope. (**A**) Red lines on the timecourse plot and bar plot mark the chance level (∼0.0004), corresponding to a two-tailed empirical estimation using 10,000 shuffled datasets, *P* < 0.05 with false-discovery rate (FDR) correction; see Analysis section for details. Arrow in timecourse plot marks response at 250 ms lag in cross-correlation. (**B**) NSE responses for individual HCs.

Figure 5 shows a parallel summary of the NSE response in these HC subjects, obtained from the same EEG recordings (see Methods section and Supplementary Figs 2 and 3). As was the case for the DPR, the average topography has multiple peaks, and there is substantial intersubject variability (Fig. 5B). However, note that the midline parieto-occipital peak seen in Fig. 4A is not present in any subject. The average temporal response is largely similar to the average DPR of Fig. 5A and will be discussed further below.

**Figure 6:**
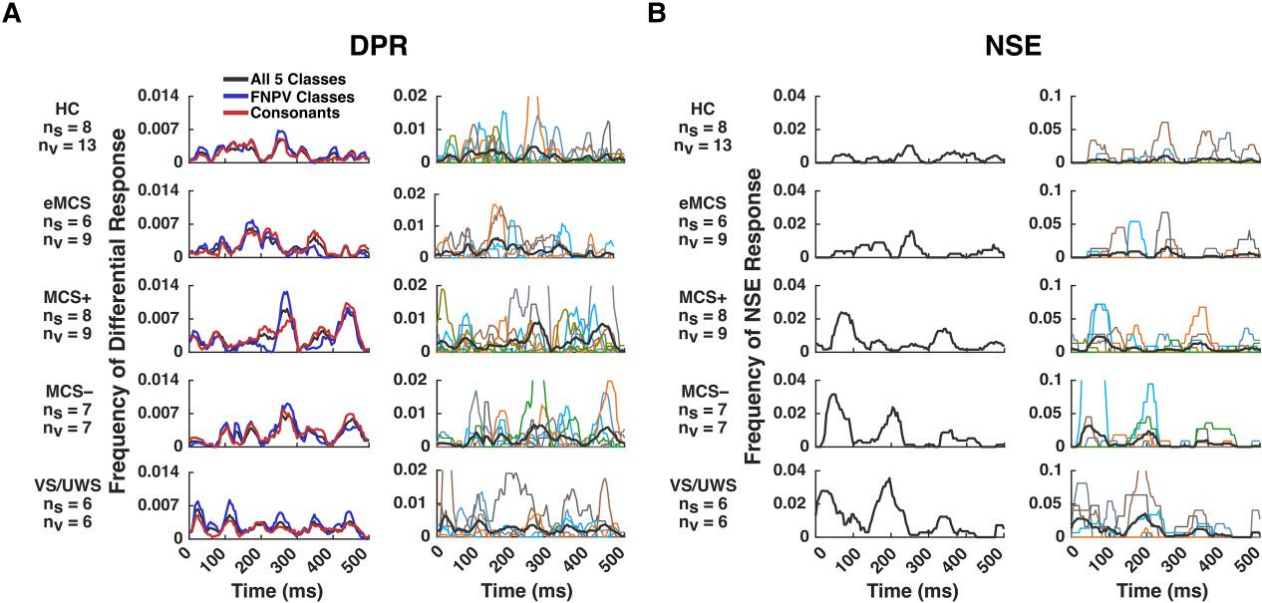
**Time course of differential phoneme-class response (DPR) and natural speech envelope (NSE) responses, categorizing patient subjects (PS) based on behavioural assessment alone.** (**A**) The top row shows the average DPR over time for healthy controls (HCs), in two ways: the left column shows the DPR averaged across pairs for all five phoneme classes (black), only consonant classes (red), and fricative, nasal, plosive, and vowel phoneme-classes (blue), for comparison with Khalighinejad *et al.;*^15^ the right column shows the DPR averaged across pairs of all phoneme classes (black, same as on the left) and for individual subjects in various colours (note difference in scale). The next four rows show results for patients grouped by behavioural classification: eMCS, minimally conscious state positive (MCS+), minimally conscious state negative (MCS−), and VS/UWS subjects respectively. n_s_ and n_v_ denote the number of subjects and visits (datasets) respectively. (**B**) Top row left shows the average NSE response for HC; top row right shows this average along with the individual responses in various colours (note the difference in scale). The next four rows show results for PS categories in the same order as **A**. The chance level is ∼0.0004, corresponding to the two-tailed Wilcoxon ranksum test for DPR and a two-tailed empirical estimation using 10,000 shuffled datasets for NSE, *P* < 0.05 with false-discovery rate (FDR) correction; see Analysis sction for details.

We now turn to the temporal aspects of the DPR and NSE responses, considering the HCs and the patient subjects (Fig. 6). As with the spatial domain, there is substantial intersubject variability for both analyses, for HC as well as all PS categories (right panels in Fig. 6A and B).

Interestingly, for the DPR (Fig. 6A), all studied patient subjects (PS) had a differential response to phoneme classes, including those who had no evidence of command-following behavioural testing. In Fig. 6A, the peak at 250 ms observed in HC is also seen in MCS+ and MCS− subjects. Additionally, MCS+ and MCS− subjects have secondary peaks at 400–500 ms post phoneme onset. For eMCS subjects, the most prominent feature is around 200 ms, somewhat earlier than the major peak in HCs. For VS/UWS subjects, the peaks do not bear an obvious correspondence to those of the HCs or the other patient categories. The two largest peaks, at 50 and 450 ms, are each driven by one subject (PS17). The peak around 120 ms is the most consistent feature, present in 3 of the 6 VS/UWS subjects (PS03, PS04 and PS14). Figure 6A also subdivides the total DPR response (black) into the subsets used by Khalighinejad *et al.*^15^ (blue) and the subgroup of consonants (red). These responses are all quite similar, and the above observations apply to these subsets as well.

For NSE (Fig. 6B), a response was also observed in all subjects, but many had only weak responses. The temporal peak around 250 ms observed in HC is also seen in eMCS, MCS−, and VS/UWS subjects. There is another earlier peak of around 80 ms in MCS+, MCS− and VS/UWS subjects. For all patient categories, each of the observed peaks is seen in multiple subjects.

**Figure 7:**
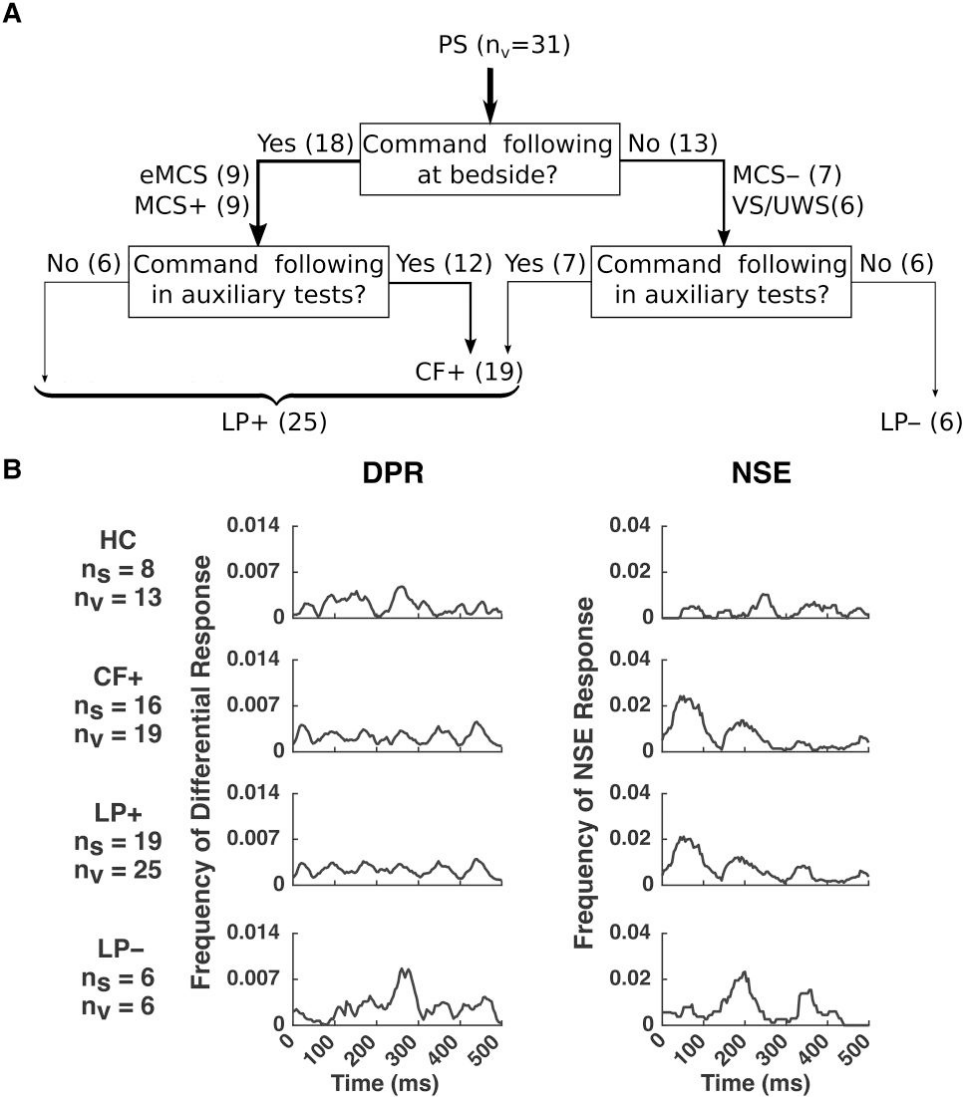
**Decision tree for recategorizing patient subjects (PS) based on behavioural, EEG, and fMRI evidence of command-following and the time course of differential phoneme response (DPR) and natural speech envelope (NSE) responses for each of the patient subject (PS) categories.** (**A**) Numbers of visits are reported in parentheses (n_v_), graphically represented by arrow thickness. Subjects with evidence of command-following based solely on EEG or fMRI (CF+) included some subjects who were classified as minimally conscious state negative (MCS−) or VS/UWS based on behavioural assessment, and not all eMCS or minimally conscious state positive (MCS+) subjects. Subjects with evidence of language processing (LP+) included all subjects with evidence of command-following in either EEG or fMRI motor imagery tasks (or both) and also subjects who had behavioural evidence of command-following, thus including all eMCS and MCS + subjects. Subjects with no evidence of language processing were classified as LP–. (**B**) Time course of DPR and NSE responses for each category of PS, based on the classification system defined in **A**. The chance level is ∼0.0004, corresponding to the two-tailed Wilcoxon ranksum test for DPR and a two-tailed empirical estimation using 10,000 shuffled datasets for NSE, *P* < 0.05 with false-discovery rate (FDR) correction; see Analysis section for details.

The results discussed above categorize PS based on behavioural assessment, which relies on intact motor function. Tests that allow independence from motor function involve steady motor imagery, which requires sustained attention. Since severe brain injury often results in loss of motor function and/or executive function, a unified method which reduces the probability of overlooking patients with language comprehension is needed. Here, we propose such a further categorization, which combines PS with evidence of command-following on behavioural assessment (eMCS and MCS+) and EEG/fMRI testing (CF+) as patient subjects with evidence of language processing (LP+). Figure 7A shows this further categorization as a decision tree. Note that LP− subjects have no evidence of language processing by any method: behavioural assessment, EEG motor imagery, or fMRI motor imagery.

Reclassifying patient subjects (PS) based on auxiliary tests of language function reveals DPR and NSE response distinctions not seen when they were categorized based on behavioural assessment alone (Fig. 7B). Specifically, PS categories with evidence of command-following had an earlier response in both analyses. For the DPR, CF+ and LP+ subjects have an early response in 30–60 ms, present in HC, but weak in LP− subjects. For the NSE response, CF+ and LP+ subjects have an early response peak of around 50 ms, absent in LP− subjects. Instead, LP− has late peaks, around 250 ms in DPR, and around 200 ms for NSE response. The responses for CF+ and LP+ subjects are similar for both DPR and NSE responses due to their large overlap in datasets (Fig. 7A).

**Figure 8:**
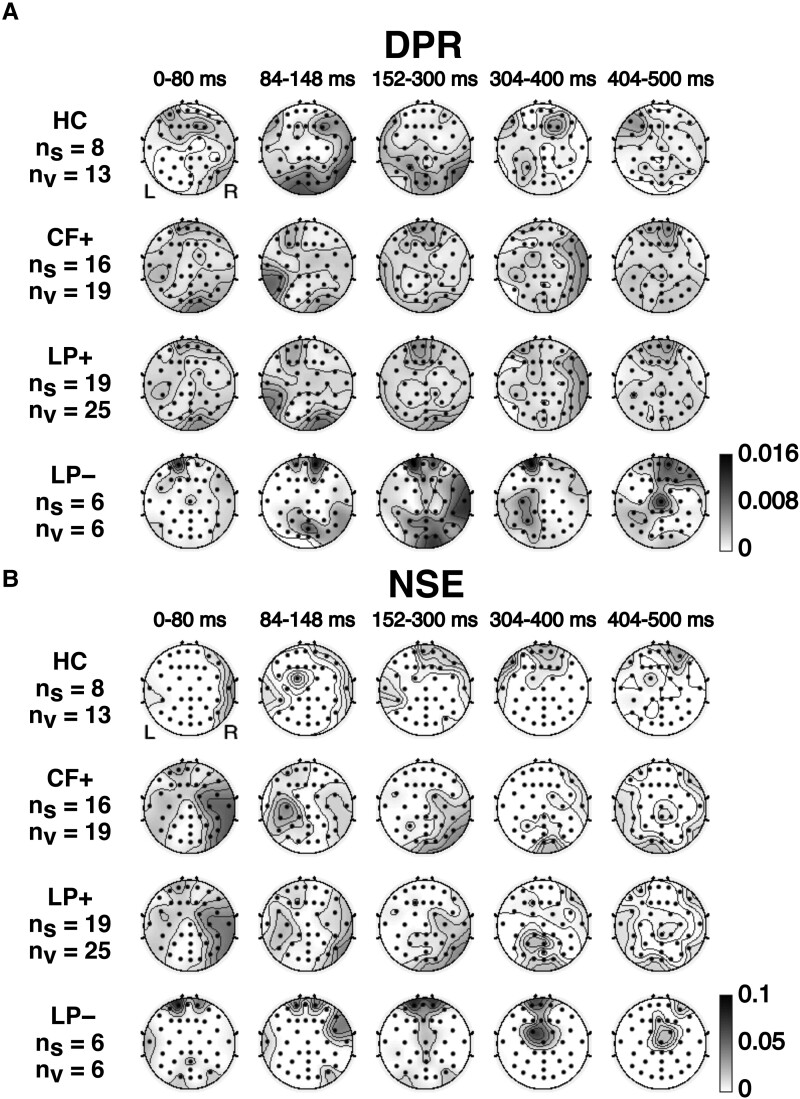
**Spatial distribution of differential phoneme-class response (DPR) and natural speech envelope (NSE) response across the analysis interval.** The 500 ms analysis interval is subdivided into five subintervals: 0–80 ms, 84–150 ms, 154–300 ms, 304–400 ms and 404–500 ms. Subjects are categorized as HC, patients with evidence of command following EEG/fMRI-based motor imagery tests (CF+), and patient subjects with and without the evidence of language processing (LP+/−). The grey levels indicate the frequency of a DPR in **A** and an NSE response in **B**. The chance level is ∼0.0004, corresponding to the two-tailed Wilcoxon ranksum test for DPR and a two-tailed empirical estimation using 10,000 shuffled datasets for NSE, *P* < 0.05 with false-discovery rate (FDR) correction; see Analysis section for details. The parallel summaries of the spatial distribution of DPR and NSE responses across the analysis interval categorizing patient subjects based on behavioural assessment alone are provided in Supplementary Figs 4 and 5.

To probe the dynamics of the spatial patterns of the observed differences in early responses, we divided the 500 ms analysis interval into five sub-intervals (0–80 ms, 84–148 ms, 152–300 ms, 304–400 ms and 404–500 ms), based on the peaks observed in responses for PS (Fig. 7B). Figure 8 shows the average DPR and NSE responses for each subinterval.

This analysis shows that the early DPR and NSE responses in CF+ and LP+ subjects are more bilateral and global than in LP− subjects (Fig. 8). The DPR responses (Fig. 8A) for CF+ and LP+ have an early occipital component as well. By contrast, LP– subjects had late DPR (mostly 152 ms and beyond) with right lateralization and no early activity in the occipital region. NSE responses (Fig. 8B) show a similar pattern: earlier and more global in CF+ and LP+ subjects than in LP– subjects, whose responses were late and confined to the frontocentral region. Categorizing subjects based on behavioural assessment alone does not show as clear differentiation within PS (Supplementary Figs 4 and 5).

**Figure 9:**
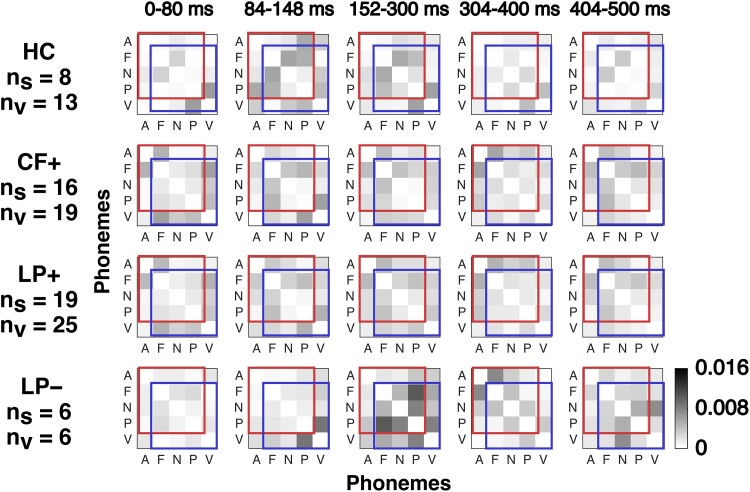
**Differentiation of individual phoneme-class pairs across the analysis interval.** Subjects are categorized as HCs, patients with evidence of command-following EEG/fMRI-based motor imagery tests (CF+), and patient subjects (PS) with and without the evidence of language processing (LP+/−). The subintervals are the same as in Fig. 8. Phoneme classes are approximants (A), fricatives (F), nasals (N), plosives (*P*) and vowels (V). R**e**sponses to each phoneme-class pair were averaged across scalp locations and trials, within each subinterval; grey levels indicate the frequency of differential phoneme-class response (DPR). The red box (covering phoneme classes A, F, N, and P) indicates the four consonant classes; the blue box (covering phoneme classes F, N, P, and V) indicates the phoneme classes used in Khalighinejad *et al.;*^15^ correspond to the blue and red traces in Fig. 6A, left column. The grey levels indicate the frequency of DPRs. The chance level is ∼0.0004, corresponding to the two-tailed Wilcoxon ranksum test, *P* < 0.05 with false-discovery rate (FDR) correction; see Analysis section for details. n_s_, number of subjects; n_v_, number of visits. A parallel summary of the differentiation of individual phoneme-class pairs across the analysis interval categorizing patient subjects based on behavioural assessment alone is provided in Supplementary Fig. 6.

Next, we looked at the dynamics of the differentiation of individual phoneme-class pairs, by studying the responses in the sub-intervals used in Fig. 8. The results are shown in Fig. 9 as heatmaps comparing all 10 phoneme-class pairs.

We found that the early differential response in CF+ and LP+ is seen for multiple phoneme-class pairs, but an early response in LP− subjects is observed only for a single phoneme-class pair (plosives versus vowels). In CF+ and LP+ subjects, differentiation of most phoneme-class pairs starts before 150 ms. In contrast, differentiation of most-phoneme-class pairs in LP− subjects starts after 150 ms.

As is the case for the contrast in the temporal and spatial organization of DPR and NSE responses between PS with evidence of language processing (LP+) and LP− subjects seen in Figs 7B and 8, the contrast in early differential responses of specific phoneme classes is lost when subjects are classified based on behavioural assessment alone (Supplementary Fig. 6). This suggests that the early response in VS/UWS and MCS− subjects is due to patient subjects who show command-following in auxiliary tests of motor function (CF+) but not in behavioural assessments. Classifying patients based on auxiliary tests identifies these subjects as LP+ subjects (Fig. 7A). The remaining subjects, i.e. the LP− subjects, do not have this early response.

Finally, we also examined the topographic maps corresponding to responses to individual phoneme-class pairs. There did not appear to be any consistent indication that individual phoneme-class pairs were preferentially differentiated in specific brain areas (data not shown).

## Discussion

This study demonstrates the use of EEG-based methods for studying lower-level language processing in DoC patients in a paradigm that does not require active engagement or sustained attention. Briefly, we use EEG differential responses to phoneme classes (DPR) and tracking of the natural speech envelope (NSE), methods that have been vetted in HCs in passive listening tasks and apply them to severely brain-injured patients.

We find that both kinds of responses are present in all DoC patient subjects studied, but those with evidence of language processing are distinguished from those without any evidence of language processing in their early responses. All patient subjects had a DPR in the first 500 ms after phoneme onset. But within this range, those with evidence of language processing in behavioural or auxiliary tests (LP+) had a stronger global response in the 30–60 ms range than patient subjects without any evidence of language processing (LP−). For the NSE, LP+ subjects had an earlier peak (∼50 ms) than LP− subjects (∼200 ms). Additionally, the early NSE response in LP+ subjects was widespread over the scalp, while the later response in LP− subjects were confined to frontal and central scalp locations. These simple responses in LP− subjects might reflect some very low-level aspect of acoustic processing.^25^

Several studies in HCs indicate that both the NSE and the DPR reflect language processing *per se* and are not merely indicators of lower-level acoustic processing. First, many studies found that acoustic processing is limited to temporal regions,^26–29^ while the DPR (Figs 4 and 8A, and Supplementary Fig. 4) and the NSE (Figs. 5 and 8B) in HCs and LP+ subjects is spatially widespread. Additionally, using magnetoencephalography, Nora *et al.*^30^ demonstrated that time-locking of the amplitude envelope of speech (essentially the NSE) is crucial for encoding acoustic-phonetic features of speech, and such temporal encoding is less prominent for non-speech environmental or human-made non-speech sounds. They also found that the cortical encoding of the speech envelope had higher fidelity than non-speech environmental or human-made sounds. Finally, as mentioned below, the early component of the DPR manifests an interaction with the previous phoneme, which also indicates that it is not merely an acoustic response.^15^

While it makes intuitive sense that impaired language function is associated with delayed responses (as we found for the NSE, see also Braiman *et al.*^16^), a more specific interpretation may be possible for the DPR. Specifically, Khaligheijad *et al.*^15^ showed that the earliest component of the DPR is distinguished from later components in that it also includes contributions related to the preceding phoneme. This suggests that the early DPR may be an electrophysiologic signature of interaction with the previous phoneme, facilitating the integration of information for higher levels of language processing. We also mention that, with sufficient data, the present method can in principle be used to identify phoneme interactions, by cross-correlating EEG responses with the classes of the current phoneme and the preceding one.

The responses in HCs are bilateral, have substantial inter-individual variability, and involve not only the left temporal lobe but multiple other regions as well. Though perhaps surprising, these characteristics are in fact consistent with previous findings along multiple lines of work as discussed below.

First, our results agree with the widely accepted dual-stream model of language processing,^31^ according to which, language comprehension is bilaterally organized in a ventral stream, extending from the temporal pole to the occipito-temporal cortices. The model further proposes that, in parallel, language production involves a left dominant dorsal stream, extending from the posterior middle temporal to the inferior frontal cortex. Additionally, recent enhancements of the model incorporate the interaction of these streams, implying the involvement of the frontal lobe in language processing.^32^

Second, although inter-individual variability has not been emphasized in previous works of phoneme processing, it is a common finding in studies of language processing based on EEG, or EEG and fMRI in combination.^33–35^ There are many theories explaining this variability in EEG responses (see Boudewyn *et al.*^36^ for review), including individual differences in working memory and linguistic experience.

Thirdly, specifically regarding the differential processing of phonemes, evidence for inter-individual variability is also found in studies aimed at decoding phonetic responses in EEG, electrocorticography, and fMRI. Though models trained across subjects have better-than-chance performance,^37,38^ better accuracy is achieved with individually trained models;^39,40^ Chang *et al.*^39^ discuss the similarities and differences in the brain regions involved in decoding phonemes. On one hand, the above-chance subjectwise accuracy of models trained across subjects indicates common language processing mechanisms. On the other hand, the wide range of accuracy reflects individual differences in processing mechanisms or their topography.

Given the variability observed between HCs, it is not surprising that PS responses are also variable, as there are further sources of variability in play, including the aetiology, extent, location(s) and duration of the injury. However, we note that in our dataset, the differences in responses reported between LP+ and LP− subjects hold across these variables.

Nevertheless, the inter-subject variability is substantial and prevents us from applying this method to individual patients at present. Further understanding of this variability is thus needed before this approach can reliably assist in prognosis and guide rehabilitation management in individual patients.

It is also worthwhile to compare our study to previous electrophysiologic investigations of assessments of residual language function in patients with severe DOC (for a general review, including fMRI measures, see Aubinet *et al.*^41^). While we are the first to our knowledge to study phoneme processing in this population (i.e. the DPR), other groups have studied the response to the speech envelope,^16^ compared the response to speech and non-speech,^42^ or investigated responses to semantics.^17,18,42^ Of these, longitudinal studies found semantic processing,^17,18,42^ but not the responses to speech and non-speech^42^, to be a good prognostic indicator. Although our study is cross-sectional, our results pertaining to the NSE and the DPR fill a gap between these studies with regard to the level of language processing. It is, therefore, consistent with the hypothesis of Aubinet *et al.*^41^ that progressive recovery of consciousness after a coma is paralleled by the reappearance of both implicit and explicit language processing.

The extent to which electrophysiologic measures of acoustic and language processing correlates with command following remains unclear. Beukema *et al.*^42^ found that VS/UWS and MCS patients could not be distinguished based on acoustic processing, and one MCS patient (but no VS/UWS patients) had an N400 response. In contrast, the current study finds that early NSE and DPR components are present in a subset of DOC patients with other evidence of command-following, but these patients, ‘LP+’, are identified not only by bedside testing (CRS-R) as in Beukema *et al.,*^42^ but also by auxiliary methods (EEG and MRI-based tests of motor imagery).

Overall, this work establishes the feasibility of low-level language processing studies in severe brain-injury patients and advances the use of EEG-based methods for the assessment of cognitive capabilities in this patient cohort. In doing so, we extend and complement the recent efforts to assay language processing in DoC patients^17,18^—which have studied the processing of words, phrases, and sentences, with short, structured stimuli—by studying phoneme processing and NSE with narrative speech. Our approach may also be applicable to responses obtained in EEG paradigms designed to assay higher levels of language processing,^17,18^ allowing for simultaneous assessment of low- and high levels of processing in this patient population.

## Conclusion

The presence of low-level language processing in patients without any evidence of command-following is a novel finding, and one that may have bearing on the prognostication and rehabilitation strategies suggested for recovery after a severe brain-injury,^2,43^ particularly those aimed at re-establishing communication, such as brain–computer interfaces.^44,45^ With the executive demands of motor imagery tasks, there is a need for cognitive tests which do not require active engagement. The innate yet complex task of language processing allows for the modular study of cognitive capabilities and is gaining prevalence in clinical studies in DoC patients.^17,18^ Application of such assessment at the individual-subject level will be an important step for personalized care and rehabilitation.^46,47^

## Supplementary Material

fcad094_Supplementary_Data

## Data Availability

The analysis was done using custom code written in MATLAB R2018b and EEGLAB package.^24^ The custom code and a de-identified HC dataset (data of HC01 shown in Figs 1–3 and Supplementary Figs 2 and 3, left panels) are available at https://github.com/jvlab/low-level_language_processing. The code takes as input the EEG, the annotations of the audio stream, and the desired groupings of phonemes into categories, and then carries out the DPR and NSE analysis described above.

## Abbreviations

CF+ =: showing evidence of command-following
CMD =: cognitive motor dissociation
CRS-R =: coma recovery scale revised
DoC =: disorders of consciousness
DPR =: differential phoneme-class responses
eMCS =: emerged from minimally conscious state
FDR =: false discovery rate
fMRI =: functional magnetic resonance imaging
HC =: healthy control(s)
LP− =: showing no evidence of language processing
LP+ =: showing evidence of language processing
MCS− =: minimally conscious state with no evidence of command-following
MCS+ =: minimally conscious state with evidence of command-following
NSE =: natural speech envelope
PS =: patient subject(s)
UWS =: unresponsive wakefulness syndrome
VS =: vegetative state
