# Supplementary material for: Low-level language processing in brain-injured patients
Source: Brain Commun. 2023 Mar 25;5(2):fcad094. doi: 10.1093/braincomms/fcad094 (PMC10088487; doi:10.1093/braincomms/fcad094)
Supplement: fcad094_Supplementary_Data [file fcad094_supplementary_data.pdf]

1.	Alice's	Adventures	in	Wonderland,	by	...
2.	ˈæləsəz	ædˈvɛnʧərz	ɪn	ˈwʌndərˌlænd,	baɪ	...
3.	ˈæləsəz	ædˈvɛnʧərz	ɪn	ˈwʌndərˌlænd,	baɪ	...

Approximants: l, r, w
Fricatives: s, v, z
Nasals: n, m
Plosives: d, ʧ, b
Vowels: æ, ə, ɛ, ɪ, ʌ, a

Supplementary Figure 1: Example of phoneme encoding of the stimulus. Line 1 contains the text in English. Vertical lines separate words. Line 2 contains the text as phonemes using the International Phonetics Alphabet. Line 3 groups these phonemes into classes according to the manner of articulation, as indicated by colors. The key shows the phonemes belonging to each class.

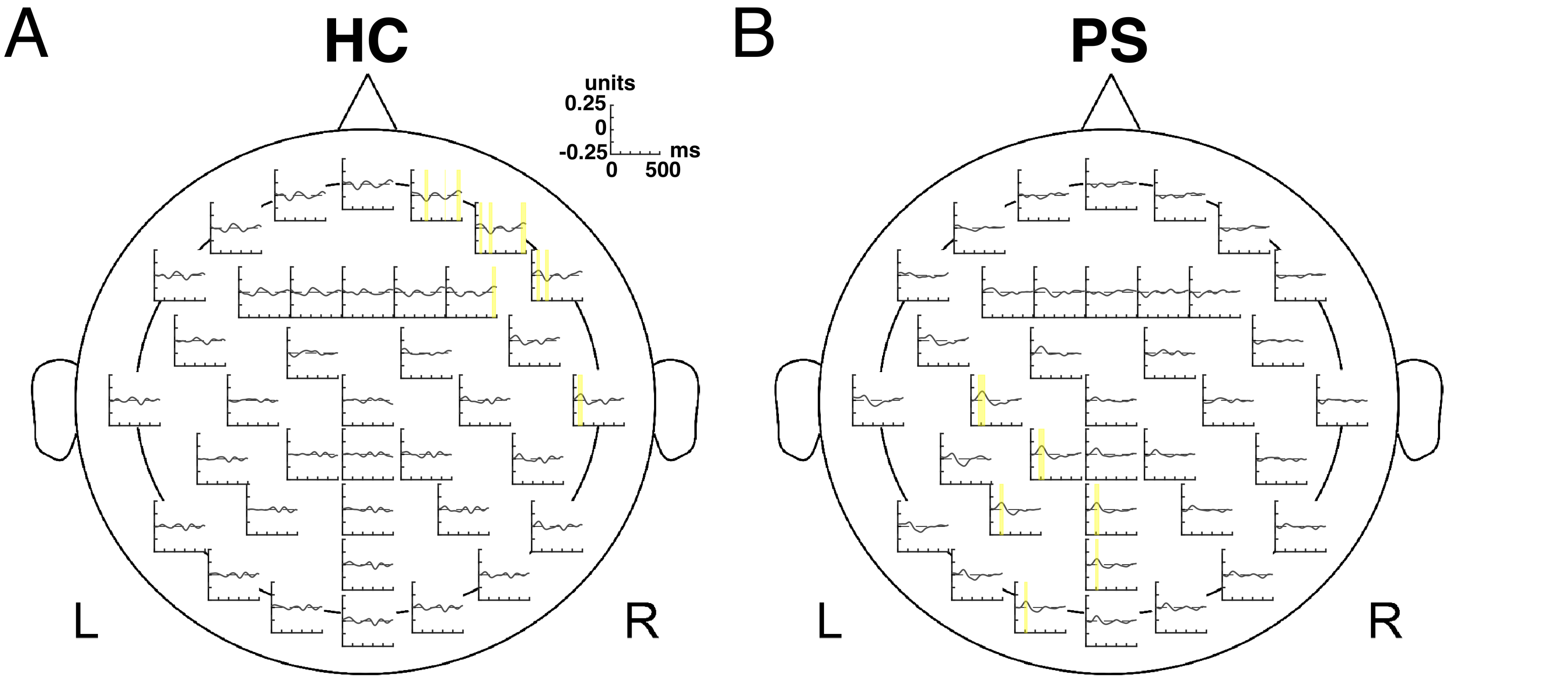

Supplementary Figure 2: Natural speech envelope (NSE) tracking response in a single trial for a healthy control (HC) and a patient subject (PS), as EEG response. For the HC (HC01) and the PS (PS11) shown in Figures 1-3 of the main text, respectively, panels (A) and (B) show, the cross-correlation of the EEG with the speech envelope for each EEG channel. Timepoints where the cross-correlation was statistically significant are shown in yellow (empirical estimation using 10,000 shuffled datasets; two-tailed, $p < 0.05$ with false discovery rate (FDR) correction; see Analysis for details).

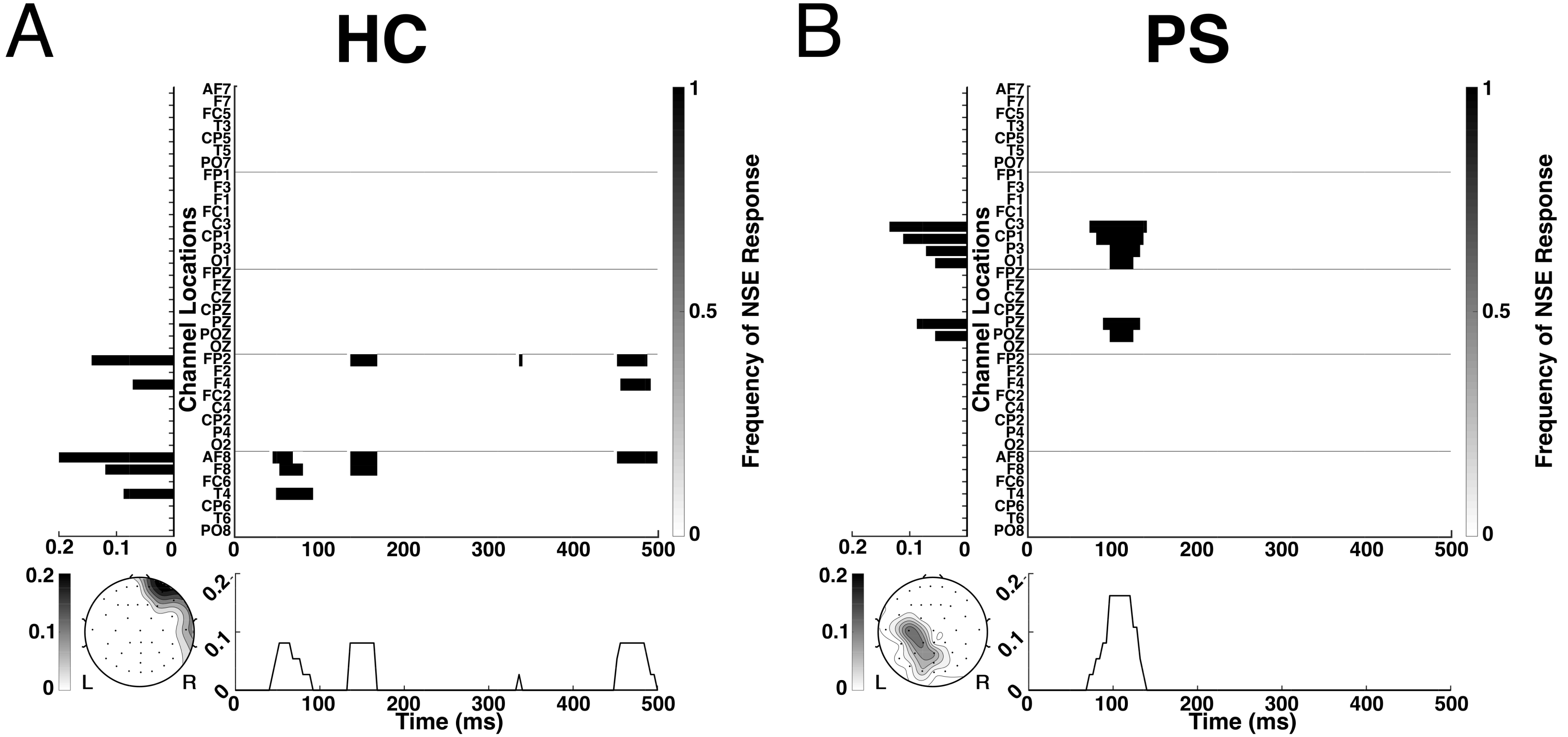

Supplementary Figure 3: Natural speech envelope (NSE) tracking response in a single trial for a healthy control (HC) and a patient subject (PS). The data in Supplementary Figure 2 is presented in a format similar to Figure 3 of the main text. The chance response level is $\sim$0.0004, corresponding to a two-tailed empirical estimation using 10,000 shuffled datasets, $p < 0.05$ with false-discovery rate (FDR) correction; see Analysis for details.

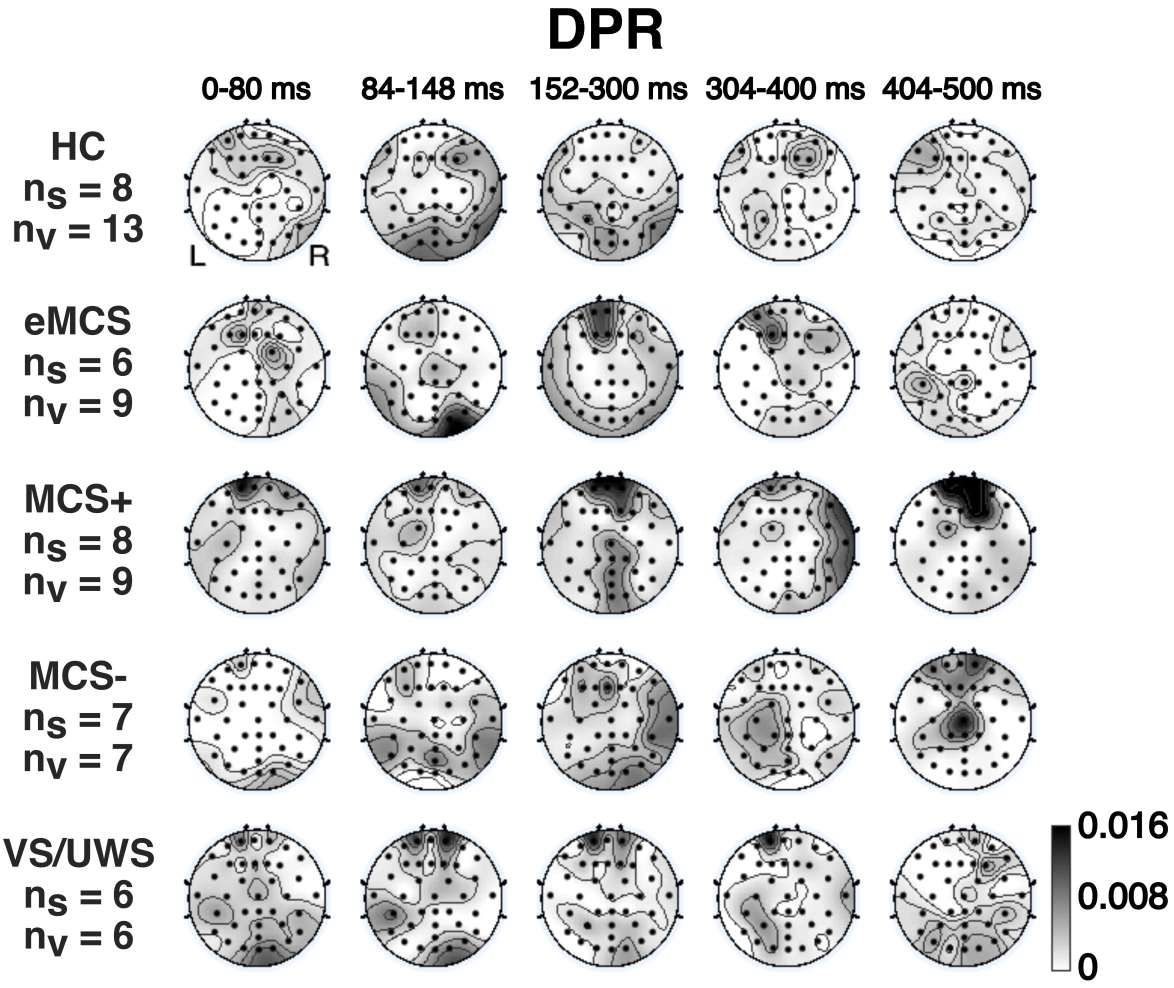

Supplementary Figure 4: Spatial distribution of differential phoneme-class responses (DPR), across the analysis interval, categorizing patients based on behavioral assessment alone. The sub-intervals (columns) are the same as in Figure 8A of the main text. The chance level is $\sim$0.0004, corresponding to the two-tailed Wilcoxon ranksum test, $p < 0.05$ with false discovery rate (FDR) correction; see Analysis for details.

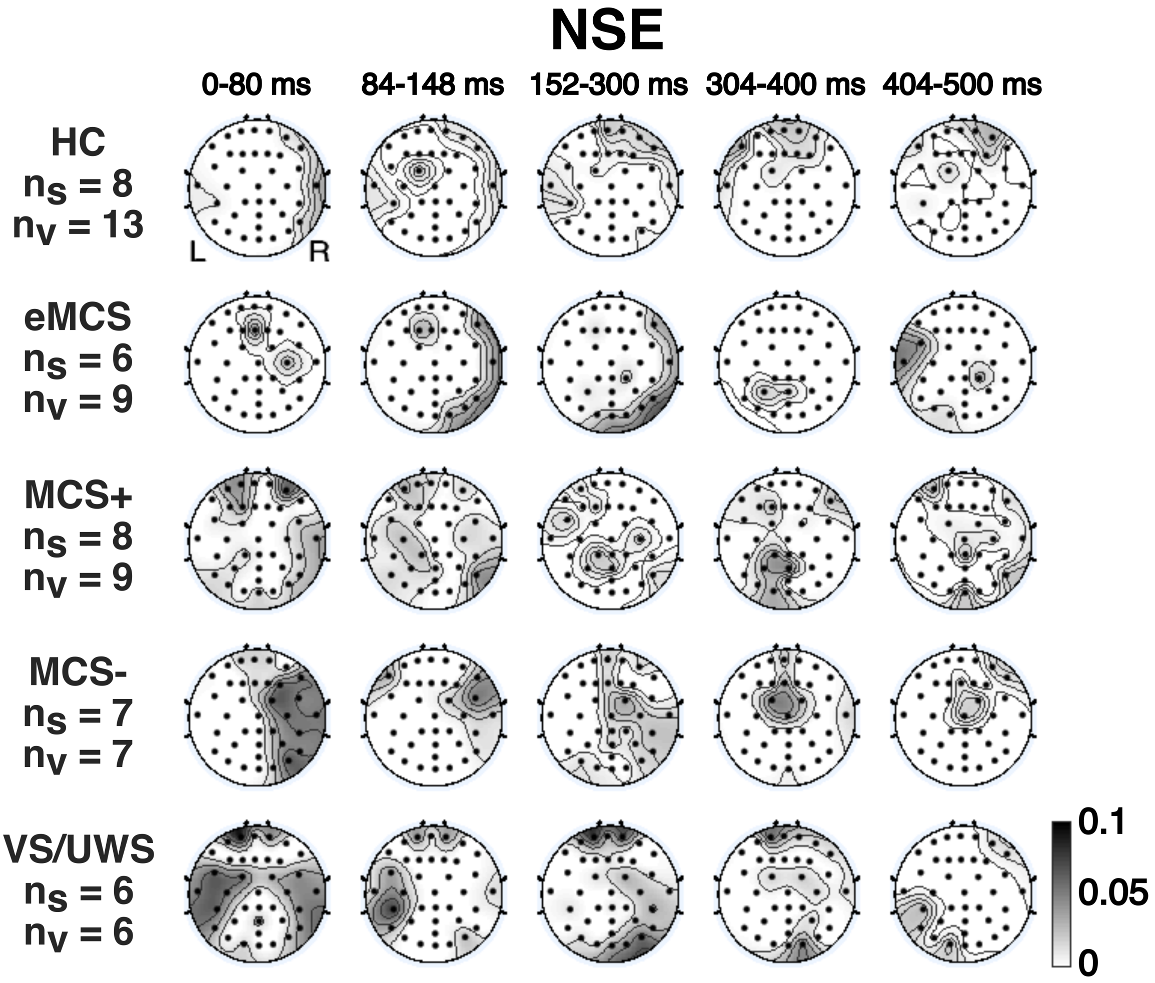

Supplementary Figure 5: Spatial distribution of natural speech envelope (NSE) responses, across the analysis interval, categorizing patients based on behavioral assessment alone. The sub-intervals (columns) are the same as in Figure 8B of the main text. The chance response level is ∼0.0004, corresponding to a two-tailed empirical estimation using 10,000 shuffled datasets, $p < 0.05$ with false-discovery rate (FDR) correction; see Analysis for details.

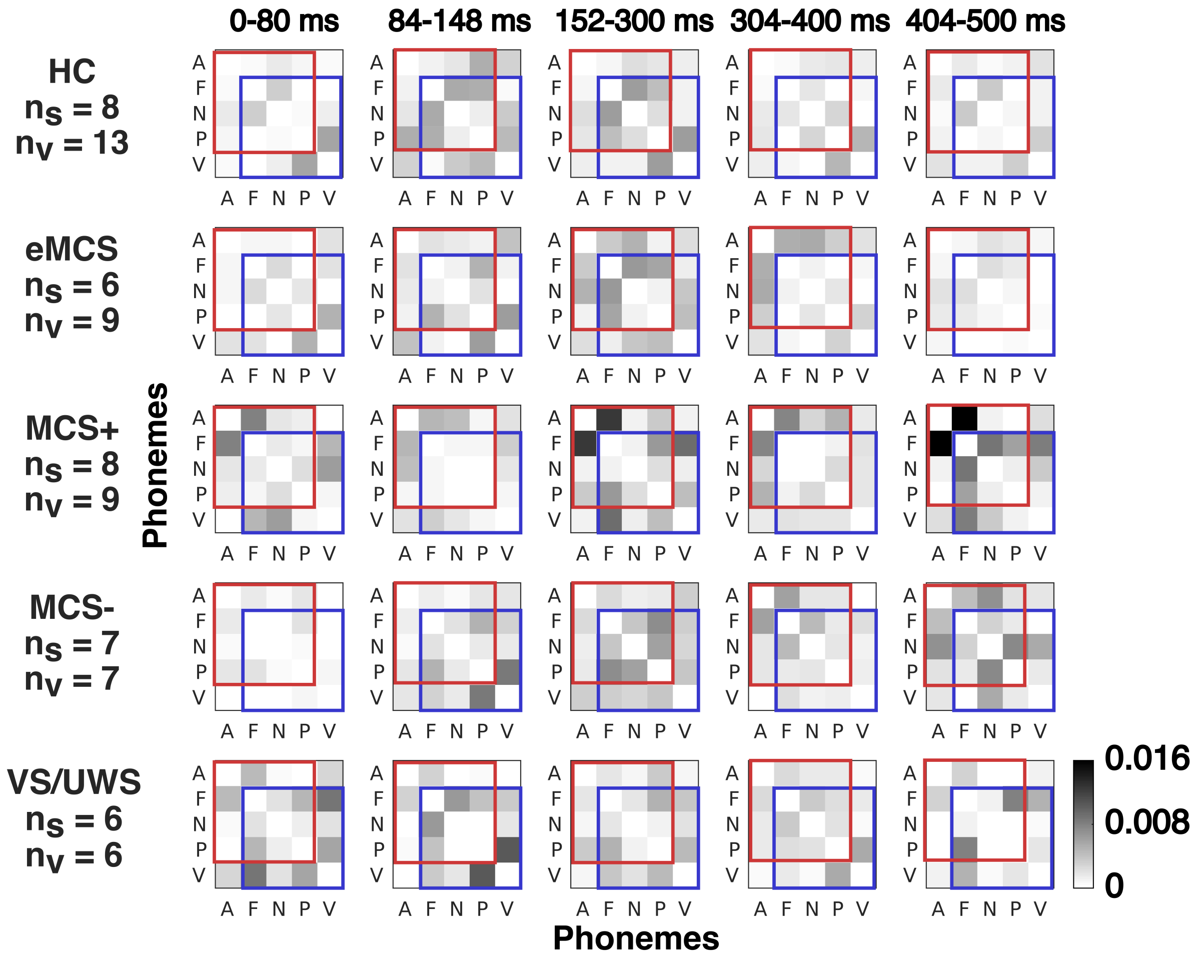

Supplementary Figure 6: Differentiation of individual phoneme pairs, across the analysis interval, categorizing patients based on behavioral assessment alone. Phoneme classes are approximants (A), fricatives (F), nasals (N), plosives (P), and vowels (V). Responses to each phoneme-class pair were averaged across scalp locations and trials, within each sub-interval; gray levels indicate frequency of DPRs. The sub-intervals (columns) are same as in Figure 9 of the main text. The blue and red boxes correspond to phoneme-class subgroups from Figures 6A and 9 of the main text. The chance level is ~0.0004, corresponding to the two-tailed Wilcoxon ranksum test, $p < 0.05$ with false discovery rate (FDR) correction; see Analysis for details.
